# The clinical utility of serum free light chain and heavy/light chain assays in monitoring disease activity in patients with IgG myeloma after achieving a deep response

**DOI:** 10.1002/ccr3.1304

**Published:** 2017-11-28

**Authors:** Kazuyuki Shimizu, Yoshikazu Kamiya, Junji Itoh, Jun Okada, Merrell Lim, Satoru Sugiyama

**Affiliations:** ^1^ Higashi Nagoya National Hospital Nagoya Japan; ^2^ Nagoya City Midori General Hospital Nagoya Japan; ^3^ Medical Biological Laboratories Nagoya Japan; ^4^ The Binding Site Group Ltd Birmingham UK; ^5^ The Institute of Applied Biochemistry Kani Japan

**Keywords:** Deep response, disease monitoring, involved/uninvolved HLC ratio, multiple myeloma, serum heavy/light chain (HLC) assay

## Abstract

Heavy/light chain (HLC) assay will enable us to evaluate the changes in the concentrations of iHLC and uHLC separately and to better identify whether the change observed is clonal or reactive. It would therefore aid in decision making for earlier implementation or discontinuation of treatment for patients with intact immunoglobulin multiple myeloma (MM).

## Introduction

It has been a consensus to utilize both serum protein electrophoretic measurement and nephelometric measurement of monoclonal immunoglobulin (M‐Ig) 1 in the disease monitoring in patients with intact immunoglobulin multiple myeloma (MM). However, monoclonal spike (M‐spike) in electrophoresis can become ambiguous when it migrates with other serum proteins such as transferrin or complement proteins. Also, in the era of novel therapies, the assessment of M‐Ig becomes more challenging because of a high incidence of attaining a deep response that will likely accompany a disappearance of M‐spike. In such clinical situations, nephelometric measurement has been recommended by guidelines 1. However, nephelometric measurement cannot discriminate between M‐Ig and normal polyclonal Ig of the same isotype. Therefore, more sensitive markers of response assessment are required.

Recently, a novel polyclonal antibody‐based serum immunoassay has been developed that separately identifies and quantifies the different heavy/light chain (HLC) types of each Ig class, providing a precise measurement of both monoclonal (involved) Ig and polyclonal (uninvolved) Ig. An important merit in clinical practice lies with the ability of this serum immunoassay to generate a HLC ratio (e.g., Ig'*κ*/Ig'*λ*) that provides an indication of clonality 2.

In this study, we closely monitored disease activity on outpatient basis using serum protein electrophoresis (SPEP), immunofixation electrophoresis (IFE), total Ig measurements by nephelometry, serum free light chain (FLC) assay, and the HLC immunoassay in patients with IgG MM who obtained deep responses composed of complete response (CR) and very good partial response (VGPR). We analyzed the clinical relevance of the HLC assay by taking all of the results and clinical information into consideration.

## Materials and Methods

Inclusion criteria for this study were limited to MM patients who achieved at least a VGPR to therapy and whose M‐Ig was suitable for monitoring by the HLC immunoassays. Therefore, patients with light chain MM and IgD MM were excluded, and because there were no patients with IgA MM who attained a deep response at our community hospital, the study cohort was limited to the patients with IgG MM only. Serial serum samples were collected at each clinic visit from six patients with IgG MM who achieved a deep response (two patients who achieved a VGPR and four that achieved a CR). Serum samples were retrospectively evaluated for HLC (i.e., IgG*κ*, IgG*λ*, IgA*κ*, IgA*λ*, IgM*κ*, and IgM*λ*), FLC (i.e., *κ* and *λ*), total Ig (i.e., IgG, IgA, and IgM), and M‐protein concentrations. Each serum sample was stored at −20°C and was thawed before testing for total Ig and M‐protein concentrations measured as in routine practice and for FLC and HLC concentrations quantified using Freelite and Hevylite reagents provided by the manufacturer (The Binding Site, Birmingham, UK) by immunoturbidimetry (SpaPlus, The Binding Site, Ltd). Written informed consent was obtained from each patient before study enrollment. The IMWG (International Myeloma Working Group) criteria were used for response assessment 3.

We have analyzed and translated the numerical data of monoclonal HLC (iHLC) and polyclonal HLC (uHLC) into a graphic form. By utilizing Microsoft Excel, we formulated a pattern of the changes of the HLC data and made it easy for clinicians to observe at a glance whether the results are indicative of an increment, decrement, or no change, as shown in the figures.

We also elaborated a schematic diagram that may help us to better understand the changes in iHLC and uHLC levels and the HLC ratio to guide patient management (Table 1).

**Table 1:** Schematic diagram of the changes in iHLC and uHLC concentrations for disease status assessment and treatment approach

Methods	Changes						
Total IgG by Nephelometry	↗	→	↘	↗	↗	↘	↘
iHLC	↗	↗	→	↗	→	↘	↘
uHLC	↘	↘	↘	↗	↗	→	↘
HLC ratio	Abnormal	Abnormal	Abnormal	Normal	Normal	Normal	Normal
Assessment	Progression	Progression	Progression	Remission	Remission	Remission	Toxicity
Action	Treat	Treat	Treat	Hold	Hold	Hold	Hold
The arrow “↓“in each figure corresponds to the respective case codes shown on the right	Case 4–1, 2, 3			Case 1–1	Case 1–2	Case 5–1	
				Case 2–1, 3			
				Case 3–1, 4			
				Case 6–1, 2			

## Sequential Changes in the iHLC and uHLC Concentrations in CR Patients

**Figure 1:**
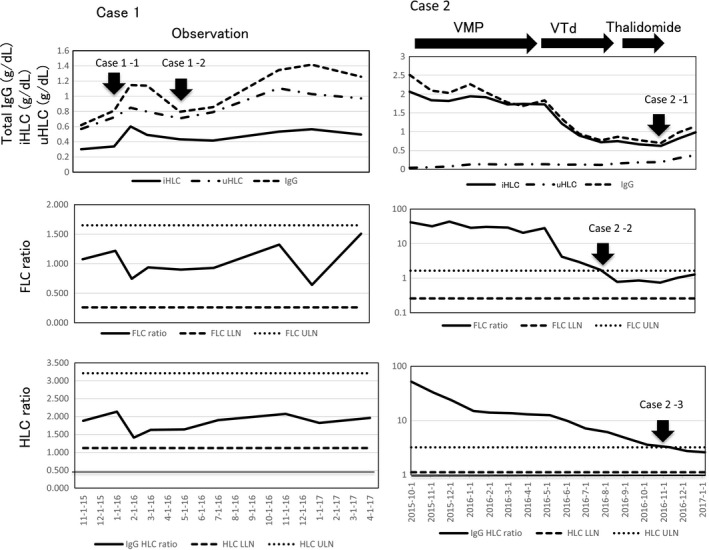
Case #1. IgG*λ* MM in CR. The increasing serum IgG levels were identified as an increment either in both of the uHLC and iHLC levels (arrow case 1–1) or in the uHLC alone accompanying a decrement in the iHLC levels (arrow case 1–2) by the HLC assay. In both occasions, the HLC ratios were normal. These HLC data were interpreted as indicating a continued remission according to the diagram (Table 1). Case #2. IgG*κ* MM in sCR. The increasing serum IgG levels were identified as an increment in both of the uHLC and iHLC levels (arrow case 2–1) by the HLC assay. The HLC ratio became normal (arrow case 2–3) and led to the identification of continued remission according to the diagram (Table 1). Each figure shows sequential changes in total serum IgG, iHLC, and uHLC concentrations (upper frame), FLC ratios (middle frame), and HLC ratios (lower frame).

Case #1 (Fig. 1): A 68‐year‐old female diagnosed with IgG*λ* symptomatic MM treated with HDM/ASCT (high‐dose melphalan followed by autologous stem cell transplantation) had attained a CR in January 2016, which has been sustained up until the patient's most recent visit in 2017. HLC assay was helpful to evaluate the disease status in two occasions indicated by the arrow case 1–1 and arrow case 1–2 in the Figure 1. In the first occasion indicated by the arrow case 1–1, a trend of increasing serum IgG levels was observed. The serial HLC assay indicated that such an increase was also seen in both of the iHLC and uHLC levels, resulting in a normal HLC ratio. In the second occasion indicated by the arrow case 1–2, a trend of slowly increasing serum IgG levels was observed, and the serial HLC assay indicated that the increment was only seen in the uHLC levels without a change in the iHLC levels, also resulting in a normal HLC ratio. All these combinations of changes in the HLC data were interpreted as indicating a remission according to the diagram shown in Table 1. Currently, her serum total IgG, IgG*κ* (uHLC) and IgG*λ* (iHLC) concentrations, and the ratios of IgG HLC, IgA HLC, and IgM HLC are all normal.

Case #2 (Fig. 1): An 83‐year‐old female with IgG*κ* symptomatic MM obtained a sCR in August 2016 as indicated by the arrow 2–2. At this time, the IgG HLC ratio remained abnormal but gradually normalized 3 months later. The abnormal ratio was considered to be due to a still suppressed uHLC and slightly elevated iHLC levels. The patient was maintained with thalidomide until November 2016. As observed in Figure 1, her serum IgG started to increase as indicated by the arrow case 2–1. However, the serial HLC assay identified a similar increase in both of the uHLC and iHLC levels which resulted in a normal HLC ratio as indicated by the arrow case 2–3. These changes in the HLC data were interpreted as indicating a remission (Table 1). Currently, her IgG HLC, IgA HLC, and IgM HLC ratios are all normal.

**Figure 2:**
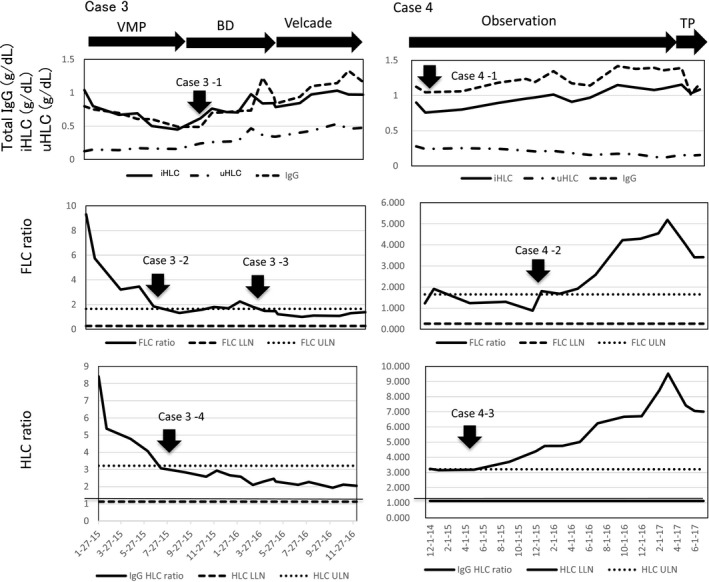
Case #3. IgG*κ* MM in sCR. The increasing serum IgG levels were identified as an increment in both of the uHLC and iHLC levels (arrow case 3–1) by the HLC assay. The HLC ratios had been normal (arrow case 3–4) and would warrant the disease activity being in remission (Table 1). The normalization of the HLC ratio occurred before the normalization of the fluctuated FLC ratios (arrow case 3–4 vs. case 3–3). Case #4. Relapsed IgG*κ* MM in progression. In spite of a normal FLC ratio, there developed an increasing serum IgG level which was identified as an increment in the iHLC accompanying a small decrement in the uHLC levels (arrow case 4–1) by the HLC assay. The HLC ratio became abnormal (arrow case 4–3) and led to the identification of relapse. The FLC ratio became abnormal (arrow case 4–2) later than the HLC ratios did (arrow case 4–3). See Figure 1 caption for sequencial changes.

Case #3 (Fig. 2): An 81‐year‐old male with IgG*κ* symptomatic MM obtained a sCR in July 2015 as indicated by the arrow case 3–2. However, his FLC ratios fluctuated and were finally within the normal range in March 2016 as indicated by the arrow case 3–3. Looking retrospectively, the patient's total serum IgG levels started to show a trend of increment around November 2015 as indicated by the arrow case 3–1. However, serial HLC assay had already identified a similar trend of increase in both of the uHLC and iHLC concentrations even before November 2015 with resultant normal HLC ratio as indicated by the arrow case 3–4. These changes in the HLC data were interpreted as indicating a remission (Table 1). Currently, IgG HLC, IgA HLC, and IgM HLC ratios are all normal. Interestingly, the HLC ratios became normal (arrow case 3–4) before the fluctuating FLC ratios did finally so (arrow case 3–3).

## Sequential Changes in the iHLC and uHLC Concentrations in a Patient Who Relapsed from CR and Progressed

Case #4 (Fig. 2): A 69‐year‐old female with IgG*κ* symptomatic MM treated with HDM/ASCT had obtained a CR. A relapse from CR was diagnosed in November 2013, but she was followed without treatment on the assumption that she was in a state similar to MGUS (monoclonal gammopathy of undetermined significance) until April 2017. Over the time period from January 2015 until March 2016, the sequential HLC assay disclosed a gradual increase in the iHLC levels along with a gradual decrease in the uHLC levels as indicated by the arrow case 4–1, implicating the development of IgG pair suppression 4. Such a change in the HLC assay resulted in abnormal HLC ratios indicated by the arrow case 4–3 and implicated a potential relapsed state according to the Table 1 in spite of the normal serum IgG concentrations as well as the normal FLC ratios. Toward early 2017, the M‐spike in SPEP gradually became more distinct, and due to the abnormal FLC ratios that had become apparent in January 2016 as indicated by the arrow case 4–2, coupled with increasing abnormal HLC ratios as indicated by the arrow case 4–3, the patient was started on thalidomide and prednisolone (TP) in April 2017. In spite of these episodes, her IgA HLC and IgM HLC ratios are normal without immunoparesis 2, 4. The HLC ratios became abnormal (arrow case 4–3) before the FLC ratios did so (arrow case 4–2), indicating an earlier relapse detection with HLC ratios. With the commencement of TP therapy, there developed an abrupt decrease in the elevated serum IgG and iHLC levels.

## Sequential Changes in the iHLC and uHLC Concentrations in VGPR Patients

**Figure 3:**
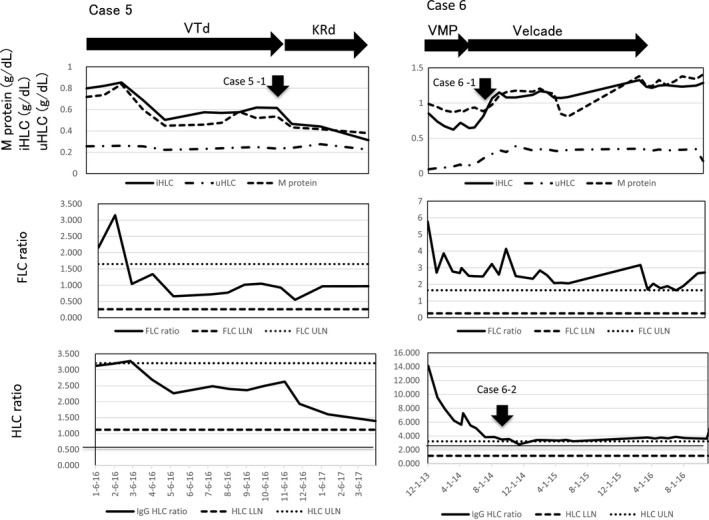
Case #5. Relapsed IgG*κ* MM. Even in a VGPR state, a favorable response to KRd was detected by the HLC assay with a steep drop in the serum M‐protein as well as the iHLC levels (arrow case 5–1). Case #6. IgG*κ* MM in VGPR. An increase in M‐protein was noted (arrow case 6–1), but the HLC assay identified it with a simultaneous increase in both of the iHLC and uHLC resulting in normal HLC ratio (arrow case 6–2). These HLC data would indicate a remission (Table 1). See Figure 1 caption for sequencial changes.

Case #5 (Fig. 3): A 75‐year‐old male with relapsed IgG*κ* MM obtained a VGPR with salvage VTd (bortezomib–thalidomide–dexamethasone) regimen. The HLC ratios as well as FLC ratios had been in the normal range since March 2016. However, due to neurotoxicity of the VTd regimen, treatment was switched to KRd (carfilzomib–lenalidomide–dexamethasone) in October 2016, with which his M‐protein levels and the iHLC levels started to decrease further without a change in the uHLC levels as indicated by the arrow case 5–1 and the patient obtained a sCR in February 2017. These combinations of the changes in the HLC data were interpreted as indicating a remission according to Table 1. However, because the patient had a relapsed disease, the KRd treatment has been continued until now. Even in a patient with VGPR, response to treatment was evaluable by the use of the HLC assay. Interestingly, his IgG HLC, IgA HLC, and IgM HLC ratios had been normal for almost 12 months prior to the attainment of sCR and remain normal until now.

Case #6 (Fig. 3): A 66‐year‐old male with IgG*κ* symptomatic MM with past medical history of cerebral infarction with resultant left hemiplegia was treated with VMP (bortezomib–melphalan–prednisolone) and obtained a VGPR which was maintained with monthly bortezomib. The patient developed a trend of increment in the M‐protein levels in July 2014 as indicated by the arrow case 6–1. However, by observing HLC trend, the HLC assay disclosed that both of the iHLC and uHLC levels were also increasing which resulted in a normal HLC ratio as indicated by the arrow case 6–2 in October 2014. These combinations of the changes in the HLC data were interpreted as indicating a remission according to Table 1. Subsequently, the patient pursued a stable clinical course in terms of symptom‐free life and normal iHLC and uHLC levels and normal HLC ratios. His physician‐in‐charge discontinued bortezomib maintenance based on the assumption that the patient had become a so‐called revert to MGUS in April 2016.

## Discussion

The value of the heavy/light chain (HLC) assay is considered to lie in its capacity of quantifying M‐Ig and polyclonal Ig of the same isotype separately in a situation where either electrophoretic or nephelometric measurements are not informative. Accordingly, this assay will enable us to evaluate the changes in the concentration of involved HLC (iHLC) and suppressed uninvolved HLC pair (designated as HLC pair suppression, e.g., suppressed IgG*λ* value in patients with IgG*κ* MM) separately, to define clonality using the HLC ratio and also to measure nonmyeloma polyclonal Ig isotypes (designated as immunoparesis, i.e., suppression of total IgA and/or IgM concentrations in IgG MM patients) 2, 4, 5.

Accordingly, the HLC assay has been expected to be of value in response assessment, disease monitoring, and prognostication of MM 2, 4, 5, 6.

In this study of a close and sequential monitoring using the HLC assay of six patients with IgG MM who had obtained a deep response, we validated findings from previous studies and became aware of several features of the HLC assay which might elicit an idea of using the HLC assay as an adjunctive test in routine clinical practice for patients with MM:

1. Because of the proven correlation between serum total Ig concentration measured by nephelometry and the sum of iHLC and uHLC levels of the same Ig isotype measured by the HLC assay 4, we were able to analyze and assess the changes in serum total Ig levels in greater detail and were able to identify whether the increase was clonal or reactive based on the calculated data of the HLC ratio 2.
  We were able to identify that an increase in serum total Ig concentration could result from two phenomena by the HLC assay: (1) In case of progression after CR as with the case #4, increase in the IgG concentration would be identified as clonal by the HLC assay by demonstrating the exclusive increase in iHLC (IgG*κ*) alone associated with a decrease in uHLC (IgG*λ*) which would then indicate the development of so‐called pair suppression 4. These changes in the HLC levels have resulted in an abnormal HLC ratio as indicated by the arrow case 4–3 in Figure 2 and led to the identification of progression according to Table 1. (2) In the context of potential B‐cell immune reconstitution after obtaining a CR, we became aware of two different phenomena in the sequential changes in iHLC and uHLC concentrations by the HLC assay: one with an increase in the previously suppressed Ig (uHLC) pair alone as in case #1 (arrow case 1–2, Fig. 1) and others with an increase in both of the iHLC and uHLC levels as in cases #1 (arrow case 1–1, Fig. 1), #2 (arrow case 2–1, Fig. 1), and #3 (arrow case 3–1, Fig. 2). The HLC ratios of these three CR patients (cases #1, #2, and #3) were all within the normal range. A study by Harutyunyan indicated that there is a significant correlation between the recoveries of uHLC toward a normal level 7.
2. In this study, MM patients who obtained normal FLC and HLC ratios after attaining CR/sCR (patients #1, 2, 3, and 4) have enjoyed their symptom‐free life for more than 19 months (#1), 12 months (#2), and 25 months (#3) until the patient's most recent visit in the clinic now, respectively, and 60 months until relapse (#4). This is in line with previous studies that have shown that after treatment normalization of the FLC ratio 8, 9, HLC ratio 2, 6, 10, and both FLC and HLC ratios 6 may be associated with a favorable outcome.
3. Although long‐term follow‐up and a larger‐scale study are needed, we have an impression that the HLC assay can be prognostic, in line with previous studies 2, 6, 10. Because in this study, patients who obtained normal HLC ratios of the affected Ig as well as normal HLC ratios of the nonmyeloma Ig were all doing well (patients #1, 2, 3, and 4). The implication of normalization of HLC ratio in nonmyeloma polyclonal Ig (residual Ig) is unknown but can be considered to represent B‐cell immune reconstitution after a successful control of tumor clone. A study by Tovar et al. in 11 concluded that a relatively higher (or normal level) HLC ratio of the uninvolved immunoglobulins is a predictor for a significant longer PFS (progression‐free survival) and even OS (overall survival) in patients with MM in CR 11. Therefore, HLC measurement of immunoparesis recovery may serve as a surrogate marker of robust immune recovery.
4. We would consider that the HLC assay could help us make decision to implement or delay treatment in patients who had obtained a deep response. In the case of minimal or no change in Ig concentrations of the same class with M‐protein, if there is an increasing iHLC level either alone or along with a decreasing uHLC level (pair suppression 4), the HLC ratio may become abnormal indicating the presence of clonality. Such changes in HLC assay may represent disease progression, and further treatment may be considered along with other relevant clinical signs of progression (Table 1). On the contrary, if there is an increase in the suppressed uHLC level alone or a simultaneous increase in both of the iHLC and uHLC levels, the HLC ratio may become normal, indicating an absence/resolution of clonality. Such phenomena may represent a B‐cell immune reconstitution and may be indicative of patient recovery. Such HLC data coupled with clinical evidence of the patient may be sufficient to deter from further treatment (Table 1).
5. There is a possibility that the HLC ratios may be predictive of remission as with case #3 (arrow case 3–4 vs. arrow case 3–3, Fig. 2) or progression as with case #4 (arrow case 4–3 vs. arrow case 4–2, Fig. 2) earlier than the FLC ratios in the close monitoring of disease activity in patients who obtained a deep response.
6. There might be another possibility that a normal HLC ratio in a patient who obtained a deep response but presenting with M‐protein and abnormal FLC ratios may implicate that the patient has become a “revert to MGUS.”

## Authorship

KS: planned and led the investigation and wrote the manuscript. YK: analyzed the results, depicted the figures, and discussed the interpretation of the results. JI and JO: did the measurements of the heavy/light chain assay. ML: analyzed and discussed the results, indicated the references, and reviewed the manuscript. SS: analyzed and discussed the results and reviewed the manuscript.

## Conflict of Interest

None declared.
